# A Treatise on Venereal Diseases of the Eye

**Published:** 1831-01-01

**Authors:** 


### VIII.

### A Treatise on the Venereal Diseases of the Eye. By *William Lawrence*, F.R.S. &c. Octavo, pp. 337. John Wilson, London, 1830.

It is not many years since the treatment of diseases of the eye was monopolized by one class of men, to the exclusion of the general mass of the profession. It cannot be denied that the breaking up of this monopoly has proved of advantage to science, to surgery, and to the public. That which is confined to the few is apt to be made a mere vehicle of gain, an engine of private interest, an instrument of quackery. The oculists and aurists have done little for the profession, but much for themselves. They have amassed wealth, but have they contributed to enlighten their brethren on what we must term the mysteries of their craft? The general voice pronounces that they have not. All reflecting men beheld with pleasure, the spectacle of ophthalmic surgery emerging from the leaden toils of those who were always inclined to secrecy and concealment, too often to undisguised charlatanerie. They prophesied that the result would be advantageous, and the prophecy has been more than amply realized. The stride in the knowledge of the diseases of the organ of vision, which has been made on the Continent and in this country, is gratifying to the philosophic lover of mankind, as well as to the practical surgeon. Destructive maladies not recognized before are now understood by every tyro, and complaints which carried on their pernicious ravages without let or hindrance from the art of medicine, are now brought under its potent control. This is no caricature, no fancy of the closet, no dream of the enthusiast. It is the simple statement of a plain fact, the proposition which is proved by daily observation. Mr. John Pearson, whose experience in venereal diseases was greater than that of any individual before or since, has declared that he never saw an instance of gonorrhœal ophthalmia, and doubted the existence of syphilitic iritis. Yet the latter are commonly met with now in any of the London Hospitals, and the former is not unfrequently witnessed wherever ophthalmic patients are collected. The conclusion is inevitable, that Mr. Pearson could not recognize these diseases when he saw them, or that patients with affections of the eye forsook the regular practitioners to throng to the professed oculists. Probably the truth will be found between the two suppositions.

We need scarcely enumerate the able men who have made the diseases of the organ of sight familiar to the reading portion of the profession. The means are well known, and the end is obvious. Ophthalmic institutions have started into vigorous existence in the metropolis, and ramifications of these parent trunks have disseminated themselves throughout the country. Mr. Mackenzie has acquired deserved reputation at Glasgow: there is an eye infirmary of no mean celebrity at Birmingham. But why should we multiply examples of what all can see, and all must applaud. We have already declared, and we here repeat it, that the results cannot fail to be beneficial to science, to surgery, and to mankind.

We hail with pleasure the work whose title stands at the head of this article. As a man of science, Mr. Lawrence cannot fail to be respected, as a man of talent, to be admired. When he dedicates his great and acknowledged acquirements to the advancement of the profession, to which he is an ornament, we are much mistaken if he will not obtain the cordial approbation of his brethren.

The work, as its name imports, treats of the Venereal Diseases of the Eye, that is, of its gonorrhœal and syphilitic affections. The mild and acute gonorrhœal ophthalmia, the syphilitic iritis, and the syphilitic ulceration of the palpebræ, are the diseases which this arrangement comprises. It must be allowed that this is an interesting field, if not a very novel one—the crop may be productive, though the grain is well known. If many of Mr. Lawrence's productions were rather doctrinal than strictly practical, rather teeming with keen remark and shrewd hypothesis than plain and sober facts, such is not the case in the present instance. There are no less than 24 cases of gonorrhœal ophthalmia—29 of syphilitic iritis—and five of syphilitic ulceration of the eyelids, all more or less circumstantially and diurnally detailed. The work may in fact be looked upon as a long-winded hospital report, which has grown fat and bulky from lying by. In the present golden age of reporting, we suppose that this will not be considered an objection to the volume.

In his advertisement or preface Mr. Lawrence declares that the account of the nature, symptoms, and treatment of the diseases which form the subject of the work, has been drawn up entirely from his own experience. Like all men of great erudition, Mr. L. is diffident of his own acquirements; he is too modest for this age of bronze. When we turn to his pages we find reference after reference to authors, English, French, and German, and he seems at least as anxious to tell us what every other person thinks, as what he himself believes. Now such is our selfishness that we had rather Mr. Lawrence had kept his word, and given us his own experience, without this alloy of foreign metal. It may indeed increase the ductility of his matériel, but it sullies its sterling worth in the assay. We are paying no fulsome compliment, and we really intend no sneer, when we affirm that Mr. Lawrence could amply afford to tell us what he knew, and to tell us that alone. We have more than enough now-a-days of the dictionary-style of writing.

The first chapter consists of an introductory and historical view of the subject, which need not detain us. The venereal diseases of the eye are naturally divided, as we said before, into the gonorrhœal and the syphilitic, and the second chapter opens with the former.

### I. Acute Gonorrhœal Inflammation of the Conjunctiva.

Three distinct forms of ophthalmic inflammation occur in conjunction with, or dependence on gonorrhœa; namely, 1st, acute inflammation of the conjunctiva; 2d, mild inflammation of that membrane; and, 3d, inflammation of the sclerotic coat, sometimes extending to the iris.

And first then of the acute inflammation of the conjunctiva; or gonorrhœal ophthalmia; or blepharophthalmia and ophthalmia gonorrhoica vera of Beer.

"The name of this affection sufficiently indicates its nature. It is a violent inflammation of the mucous membrane of the eye-ball and lids, attended with a profuse discharge of fluid, closely resembling in all its sensible characters that, which issues from the inflamed urethra in gonorrhœa, and occurring in some kind of connexion with that complaint. It is the most violent and rapidly destructive inflammation, to which the eye is subject; and, fortunately, it is one of the most rare. It sometimes destroys the eye within a very short time; and the organ is often irreparably injured before the patient seeks for surgical relief, especially when the affection occurs in the lower classes.

Purulent ophthalmia, in its various forms, commences in, and is at first confined to the mucous membrane of the eye. It soon extends to the cornea, which it either disorganizes, or changes in structure so considerably, as frequently to destroy or seriously injure sight. The whole texture of the inflamed conjunctiva swells; its blood-vessels are distended to the highest degree, and the membrane becomes of an intense bright red. The texture of the mucous surface is loosened and softened—it becomes pulpy and granular; in short, very much like the secreting surface of some parts of the alimentary canal; and, in this altered state, it pours forth the puriform discharge, which is not produced by suppuration, but is merely the ordinary mucous exhalation, altered in its qualities, and increased in quantity by the inflammation of the membrane. Indeed, purulent ophthalmia does not in general produce suppuration: the changes, which it more commonly causes in the cornea, are sloughing, ulceration, and interstitial deposition ending in opacity." 12.

*Symptoms and Progress of the Complaint.* This presents all the characters of purulent inflammation of the conjunctiva in their fullest extent. In the first short stage, the inflammation is confined to the conjunctiva, and is attended with soreness and stiffness, the sensation of sand or dirt in the eye, and more or less uneasiness on exposure to light or using the organ. It soon extends to the cornea, with agonizing pain in the globe, orbit, and head, aggravated by exposure to light, and attended with inflammatory fever. When the disease has advanced thus far, we can hardly expect, by any kind of treatment, to avert entirely its destructive consequences. The violent inflammation, which produces the puriform discharge from the surface of the conjunctiva, gives rise to effusion into the cellular texture connecting it to the surrounding parts. Hence the general swelling of the membrane, and especially the chemosis round the cornea, sometimes so considerable as to overlap and nearly hide it. A similar effusion takes place into the cellular texture of the eye-lids, particularly the upper, which sometimes hangs over and completely covers the lower. This swelling is sometimes simply œdematous; at others, it is firmer, and the skin, particularly of the upper lid, is of a bright red. This denotes more active inflammation and greater danger to the organ. The chemosis and palpebral swelling make it often difficult, sometimes impossible to obtain a clear view of the cornea, and we should not persist in such efforts at the risk of aggravating the inflammation. In the progress of the case the œdema of the lids declines, and then one or both may become everted, the convex edge of the tarsal cartilage being pushed forwards by the swollen conjunctiva.

The chemosis and tumefaction of the eye-lids are obviously analogous to the effusion in the neighbourhood of any active inflammation. Yet such modern writers as Beer and Richter profess the old opinion, that they depend on the effusion of true gonorrhœal matter. Richter gravely affirms that this flows out on making incisions into the chemosed conjunctiva.

The inflamed membrane at first exhales a small quantity of thin whitish mucus; as the inflammation proceeds, the discharge becomes thicker, yellow, and abundant; its quantity and yellowness being proportionate to the inflammation. When the latter is at its height, the discharge closely resembles, in appearance and its stain on linen, the gonorrhœal discharge from the urethra.

Although the pain in the eye and head is sometimes very severe, the globe feeling as if it would burst, yet there are some instances in which there is little or no suffering. As might readily be anticipated, the symptoms of acute gonorrhœal ophthalmia are not equally violent throughout its course. The affection may be divided into three stages. In the first, there is vascular distention and swelling of the membrane, with swelling of the lids: in this there is some pain in the organ;—in the second, there is puriform discharge, with increased uneasiness;—in the third, there is extension of the inflammation to the cornea, with great aggravation of suffering. The duration of each stage varies with the condition of the individual, and perhaps still more according to the nature of the treatment. The variety is greatest in the third stage, the two first passing off very rapidly. In Case V. the second stage began in twenty-four hours from the first perception of uneasiness; in thirty-six hours the eye could not be seen, and the disease had clearly extended to the cornea. In Case I. the first stage occupied about thirty-six hours, the second between three and four days. In Case VIII. the affection began on the 1st of November, and on the 6th the cornea was found to have sloughed. These facts exemplify the frightful rapidity of the disease.

*Effects of the Inflammation.* The immediate effects upon the cornea are sloughing, suppuration, ulceration, and interstitial deposition. The more remote consequences are escape of the humours and collapse of the globe, obliteration of the anterior chamber and flattening of the front of the eye, staphyloma, prolapsus iridis, obliteration of the pupil, corneal opacity, and anterior adhesion of the iris. The eye is sometimes said to *burst.* A paroxysm of excruciating pain is suddenly terminated by a sensation of something giving way, a little fluid runs down the cheek, and great relief is experienced. The anterior chamber has been penetrated in consequence of the changes of the cornea.

The cornea becomes dull and hazy before it sloughs, indeed, before any of the preceding changes have occurred. When it has sloughed it is converted into a dirty yellowish or brownish opaque substance, which at first looks like a portion of wetted leather, but is soon separated from the living parts, when it has a loose, soft, and ragged appearance. As this separation exposes the transparent lens and capsule, the patient sometimes recovers, for a short period, tolerably good vision. After the slough is detached, the chambers of the aqueous humour may be exposed by ulceration ; the humours will then escape, the emptied coats will collapse, and the globe remains permanently shrunk in the socket. More commonly the entire thickness of the cornea does not separate, and the anterior chamber is not exposed. The interior layer of the cornea, or the membrane of the aqueous humour is left, and is soon pushed forwards by the iris, which forms an irregular, brownish, and dirty-looking protuberance in the front of the eye. As the inflammation declines this protuberance recedes until it disappears altogether, the front of the eye remaining flattened, and being formed by the iris, covered by a thin, smooth, and more or less opaque pellicle, through which the fibres of the iris may be distinctly seen, giving it a somewhat streaked appearance. Sometimes the iris is permanently protruded, and forms a dark, more or less smooth protuberance, partially subdivided on the surface, and constituting *staphyloma racemosum.*

The separation of the slough, when partial, leaves an ulcerated surface, which is soon raised into a vesicular protuberance, consisting of the membrane of the aqueous humour, with the iris which has become adherent during the previous inflammation of the cornea. This forms *prolapsus iridis.* It shrinks as the inflammation declines, and the regular figure of the cornea is restored ; but the iris remains adherent, and is covered only by a thin pellicle, which is partially opaque, while the boundary of the adhesion presents a deeper opacity in the cicatrix of the corneal laminæ. If as much as one-half or one-third of the cornea should have perished, a permanent tumour is sometimes formed in the front of the eye, consisting externally of the opaque cornea, and internally of the adherent iris; its cavity, which is an extension of the anterior chamber, being filled with the aqueous humour. This is termed partial staphyloma, and differs from prolapsus iridis, or complete staphyloma, only in size. Mr. Lawrence has seen this occur in both eyes of the same individual, with but little injury to sight, as the protrusion of the iris hardly interfered with the pupil.

Suppuration of the cornea may be general or partial: it is usually the former. The cornea first becomes white, and then assumes a yellow colour. The effused substance is not a fluid, nor collected into a cavity; it is a thick viscid matter deposited in the texture of the cornea. Ulceration takes place, and exposes an opaque yellow substance, which looks like ordinary matter, but cannot be wiped off. The ulcerative process extends until this is removed. If the whole cornea is destroyed the humours may escape, and the globe will shrink. Or, the humours may remain, and the tumid conjunctiva scleroticæ contract from the circumference towards the centre of the space left vacant by the cornea, until it completely fills it; the eye then appears like a red fleshy mass, in which even the original situation of the cornea cannot be distinguished. The ulceration of the suppurated cornea may penetrate the anterior chamber at different parts, at each of which the iris may protrude, the front of the organ remaining ultimately flattened.

When ulceration takes place without previous suppuration, it generally attacks the margin of the cornea, and extends rapidly through the laminæ, forming a deep trench, seldom occupying less than one-third, often one-half or two-thirds, of the circumference, and sometimes extending round the whole circle. In the latter case the portion insulated by the ulcerative process sloughs. On the sides of this ulcerated trench, the laminæ of the cornea may be seen very distinctly. Beer compares them to the leaves of a book, which turn up after it has been much read. If the ulceration occupy no more than two-thirds of the margin, the vascular supply of the cornea will still be carried on, and the mischief may be repaired. As the margin of the cornea is covered by the swollen conjunctiva, we do not see the ulcers until the chemosis begins to subside. When the ulcer has gone through the corneal laminæ, the membrane of the aqueous humour may rise as a transparent vesicle in the cavity; or it may be pushed forwards by a protruding portion of the iris. The ulcerative process may penetrate the anterior chamber, when the iris will either fall against the opening, or be pushed into it and block it up. If the ulcer, however produced, should be spreading, the inflammation remaining unchecked, its surface is whitish and ragged, or flocculent; or of a dirty, yellowish cast, with surrounding haziness. When the inflammation subsides, it becomes transparent. The commencement of the restorative process is marked by the surface of the excavation assuming a light greyish tint, with a jelly-like appearance. A soft semi-opaque substance slowly fills up the breach, when the surface becomes smooth, and the regular figure of the cornea is restored. No secretion of pus is observed, either during the stage of ulceration or that of reparation. The latter process is slow, several days often elapsing without any sensible change in the size or appearance of the ulcer. The same contraction takes place here, as after the cicatrization of other ulcers, so that the size of the opaque cicatrix is less than that of the previous corneal ulcer; one of considerable size leaving a mark that is scarcely perceived, whilst, although the ulceration may have extended over the edge of the pupil, the cicatrix may leave that aperture quite unobstructed.

The existence of ulceration makes no difference in the pain during the active period of the disease: when the inflammation is stopped, a large ulcer may give little pain. When interstitial deposition takes place in the corneal lamellæ, the opacity is of the dense kind called *leucoma* or *albugo*. It is usually accompanied by anterior adhesion of the iris, or *synechia anterior.* Contraction or obliteration of the pupil may occur, in consequence of the protrusion of the iris in partial staphyloma, or at the smaller apertures produced by ulceration; or of its adhesion to a leucomatous portion of the cornea. When the cornea has been weakened by extensive sloughing or ulceration, the iris having previously become adherent to it during the active period of the inflammation, the conditions necessary to the formation of total staphyloma exist; which accordingly is one of the ultimate consequences of gonorrhœal ophthalmia.

*Diagnosis.* The peculiar nature of gonorrhœal ophthalmia is indicated only by existing or preceding gonorrhœa. In general, it attacks only one eye, while purulent ophthalmia affects both. Ordinary purulent ophthalmia may, however, be confined to one eye, whilst the gonorrhœal form often attacks the second eye after a short interval. One eye only was affected in nine out of the fourteen cases which Mr. L. has recorded. In common purulent ophthalmia, the mucous lining of the palpebræ is first affected, and the disease extends to the mucous covering of the globe. In Case V, where the complaint was seen at its outset, it began at once in the conjunctiva oculi. It is further characterized by the violence of its symptoms, the rapidity of its progress, and the short time in which it produces destructive effects. The few scattered cases of purulent ophthalmia met with in civil life are much less alarming in these respects.

*Prognosis.* In a large proportion of these cases, the sight is either lost or seriously injured. Out of fourteen cases, loss of vision took place in nine from sloughing, suppuration, or opacity of the cornea. In two of these one eye was lost, and the other recovered. Sight was restored in the other five, with partial opacity of the cornea, and anterior adhesion of the iris in three of the number. Our assistance is seldom obtained in the early stage, and even if it is, success is far from invariable. If the cornea should possess its natural clearness, the eye may be saved. If it is hazy and dull, and particularly if it has assumed a white nebulous appearance, more or less serious consequences will inevitably follow. Great swelling of the conjunctiva, especially great chemosis, profuse yellow discharge, and bright redness of the swollen upper eyelid, are unfavourable circumstances. The changes of the cornea do not always destroy sight, which may be restored after its partial sloughing, and may not be injured by extensive ulceration. All cases are not equally severe. When both eyes are attacked in succession, the disease is less severe in the second, which, therefore, is usually saved. Sometimes the inflammation is equally violent and destructive in both. Mr. Lawrence has seen more than one melancholy instance of total blindness.

*Causes.* "In investigating this part of the subject, we have to inquire what is the nature of the connexion between this inflammation of the eye and gonorrhœa? whether the former can be produced by the application of gonorrhœal matter to the organ? If so, whether an individual can infect himself? whether the application of matter from another source be necessary? or whether the infection may occur in both ways? Whether, on the other hand, gonorrhœal ophthalmia may be an example of that peculiar transference of diseased action, which is called metastasis? To some it may appear necessary to examine a previous question; viz. whether there is any connexion at all between the inflammation of the urethra and that of the eye? For Mr. PEARSON, in a letter which I have already quoted, directly denies the existence of such a connexion, on the ground that, in many thousand cases of gonorrhœa, he had not seen one instance of inflammation of the eye that could be ascribed to the gonorrhœa.

From this statement we can merely infer that Mr. PEARSON had not seen gonorrhœal ophthalmia, which is very strange, when we consider, as he informs us, that many thousand cases of gonorrhœa had fallen under his notice. I may oppose to his negative testimony the positive experience of many competent observers, and the evidence of the facts detailed in this work.

Whether this dangerous ophthalmia can be produced by the application of gonorrhœal matter to the organ, is a more doubtful point, which the nature of the subject prevents us from settling in the only satisfactory way, that is, by direct experiment.

It is stated incidentally by BEER,* and in the same kind of way by SCARPA, that, if gonorrhœal matter be applied to the eye, it excites only a slight degree of inflammation. These statements are not accompanied by any narratives of cases, or other detailed illustrations, so that we do not know on what kind of proof the assertions rest, nor how the application of the morbid secretion to the eye was ascertained." 29.

Infectious matter does not produce disease in the same individual, and analogy would lead us to imagine that the law held good with gonorrhœa. Dr. Vetch took matter from the eyes of persons labouring under acute purulent ophthalmia, and applied it to the urethra of the same individual. No disease was excited. He applied it to the urethra of another individual, and a very virulent gonorrhœa was the consequence. But morbific influences are not in all instances reciprocal, and the converse of Dr. Vetch's experiment might not succeed. Dr. Vetch mentions that a hospital assistant, named Smith, applied gonorrhœal matter to his own eyes with impunity, and no doubt it is often brought in contact with the eye in the lower classes, yet gonorrhœal ophthalmia is comparatively rare.

"Although these various considerations would lead us to expect that gonorrhœal discharge would not affect the eyes of the same individual, we meet in practice with cases, from which there is every reason to draw the contrary conclusion. It is a well known popular remedy for sore eyes to wash them with one's own urine; and persons labouring under gonorrhœa are sometimes so thoughtless as to resort to this practice. Experience teaches us that this direct application of infectious matter is capable of producing, not such a slight inflammation as BEER and SCARPA speak of, but acute gonorrhœal ophthalmia in its most destructive form. This is fully proved by CASE IV. where both eyes were lost, and CASE XIV., in which the vision of one was destroyed. In CASE VIII., in which partial sloughing of one cornea occurred, the patient had used to his eyes a towel soiled with gonorrhœal discharge from his own urethra. Mr. WARDROP communicated to me two cases, which occurred under his own observation. In one of them, that of a young gentleman labouring under gonorrhœa, who had inadvertently touched his eyes when his fingers were contaminated with the discharge, violent puriform ophthalmia occurred, and ended in the suppuration and collapse of both eye-balls. A soldier, who had gonorrhœa, was advised to bathe his eyes with his own urine, as a remedy for a slight affection of the lids: purulent ophthalmia seized one eye, which suppurated and

* "'We read of gonorrhœal ophthalmia said to have been produced by infection, that is, by gonorrhœal matter of the same or another individual conveyed to the eye by a cloth, or the fingers. I have frequently seen such cases, but they were merely inflammations of the palpebral glands, which yielded to the ordinary means. It is possible, however, that the inflammation of the palpebral glands, thus produced by immediate infection, might pass into purulent inflammation of the eyelids and eye in a weakly person of bad constitution.'—*Lehre*, v. i. § 540."

burst. ASTRUC* saw a case in which both eyes became inflamed from this cause; but the affection does not seem to have been very severe. Another instance is detailed by Mr. FOOT;† the ophthalmia, which was of the most acute kind, ended in opacity of the cornea and loss of vision.

Experience clearly proves, what we should have expected a priori, that gonorrhœal ophthalmia may be produced by the application of gonorrhœal matter from another individual. This cannot be a very frequent occurrence for obvious reasons; and I have seen no instance of the kind. Mr. WARDROP has furnished me with two examples. An old lady went into the dressing-room of her son, who had gonorrhœa, and washed her face with a towel which he had been recently making use of. Purulent ophthalmia quickly supervened, and destroyed the eye in a few days. A washerwoman, who had been employed in cleansing foul linen, was seized in a few hours with puriform ophthalmia, which terminated in the suppuration and collapse of both eyeballs. DELPECH‡ mentions the instance of a young and healthy woman, who washed her eyes with goulard water and a sponge, which had been used by a young man affected with gonorrhœa. Violent ophthalmia came on, and quickly terminated in loss of the eye. Mr. BACOT§ distinctly traced the origin of the disease to infection, by means of matter from another individual, in three instances, two of which were washerwomen." 33.

In a great proportion of cases we cannot trace the disease of the eye to the application of infectious matter. The cause is said to be metastasis and the suppression of the urethral discharge. Mr. Lawrence, however, has never seen the discharge cease, and although diminished it still continues. On the other hand, the sudden stoppage of gonorrhœa, when effected by surgical treatment, is not followed by inflammation of the eye. Mr. Lawrence is inclined to refer the occurrence of gonorrhœal ophthalmia to a peculiar state of the constitution, analogous to what is seen in gout and rheumatism. He thinks that this view is borne out by the fact, that the affection is much more common in males than in females. Besides, the morbid influences experienced and exerted by the male urethra are different from those of the vagina.

*Treatment.* The only chance of saving the eye is afforded by the boldest antiphlogistic treatment, particularly local and general depletion. We must bleed largely from the arm, and take blood by cupping on the temples, or by numerous leeches applied round the part; these measures must be repeated at short intervals until the vascular congestion is relieved and the pain removed. The other items of the antiphlogistic treatment must be added. If depletion has failed in some of his cases, Mr. Lawrence believes that it is not because it is ineffectual, but because it was not carried to a sufficient extent. Mr. Wardrop informed our author that in the only instance in which he had seen the eye preserved, the patient, a young woman, lost 170 ounces of blood in a few days, and her skin had the hue of a wax candle. In the cases recorded by Mr. Lawrence, in which the termination was most favourable, blood was taken very largely.

For the slighter symptoms which may shew themselves after the inflammation has been subdued, local bleeding will suffice. The bolder depletion is recommended where the inflammation is fully developed, without the cornea being yet affected, or where the condition of the cornea may be

* Vol. i. p. 295. † Treatise on Lues Venerea; 1820, p. 98.

‡ Chirurgie Clinique; t. 1, p. 318. § Treatise on Syphilis, p. 132.

doubtful; that is, where we may entertain the expectation of saving the organ from all injurious change.

If sloughing or suppuration should have occurred already more moderate depletion is indicated. General sloughing or general suppuration of the cornea is usually attended by diminution of the inflammation and cessation of pain, or at least comparative ease. The loss of blood is not required for the suffering and cannot preserve the vision. After partial sloughing the inflammation may still continue unabated, and active depletion may still be required both to limit the extent of the mischief, and to favour the process of separation and restoration. In one case this treatment was perfectly successful in preserving sight. Blisters are only to be looked on as an auxiliary measure, to be resorted to after other antiphlogistic means. They should be applied to the back of the neck, and the discharge should be kept up by the savine cerate. Some recommend hot applications, others cold ; neither are possessed of any great power. Cold are most applicable to the early stage, but the patient's feelings must be our guide. A saturnine lotion made with rose-water or poppy-fomentation is useful. The eye-lids and cheek should be frequently cleaned, and the discharge prevented from encrusting on the lids ; these may sometimes be smeared with some mild unctuous substance.

The inflammation may be checked by these means, but its effects are not immediately removed. The conjunctiva grows paler and somewhat flabby, and the purulent discharge is still abundant. The patient also is probably pale and weak. It is now commonly considered necessary to give tonics internally and employ astringents locally. If the inflammation has been speedily and completely subdued, mild aperients alone will effect the cure, with the addition perhaps of a weak sulphate of zinc lotion.

Occasionally, but very rarely, sloughing of the cornea is attended with a change of symptoms, and general depression requiring cordials and tonics. Whilst this occurs, the ulcer on the cornea may spread with a whitish or dull yellow colour, and an irregular surface and edge. Here bark and a generous diet are necessary. Astringent lotions are also proper, and whenever the inflammation has been subdued they may safely be employed; although they may not accelerate recovery, they will not, under such circumstances, materially retard it. Sometimes, however, they act as stimuli and induce a relapse. Mr. Lawrence thinks this will be most certainly averted by avoiding all local excitement, and pursuing mild antiphlogistic treatment. The best forms of astringent applications are the solution of alum, gr. ij. to gr. x. to the ounce of water : the solution of the nitrate of silver : and the undiluted liquor plumbi. Means of this kind, and especially the lunar caustic, have been recommended in ulcers of the cornea, particularly when accompanied with protrusion of the iris ; in the latter case it has been used in substance. Mr. L. has found recovery take place most speedily when none of these means have been employed.

The use of a strong injection has been recommended in the very commencement of the affection. Mr. Melin proposed it in ordinary inflammation of the conjunctiva, dropping a solution of four grains of the nitrate of silver to the ounce of water, into the eyes twice a-day ; in a few days the cure was effected. Mr. Melin has tried this plan very extensively in acute ophthalmia, and has amply proved its efficacy. Dr. Ridgway, with whom this practice originated, employed a solution of ten grains to the ounce, and used it in gonorrhœal as well as conjunctival inflammation. Dr. O'Halloran has advanced the strongest testimony in favour of the astringent plan of treatment in ordinary purulent ophthalmia. He employed the sulphate of copper in substance, or dropped into the eye the ten-grain solution of the nitrate of silver, generally using one or the other once a day. He gave purgatives and applied fomentations, with leeches, if there were symptoms of affection of the internal parts of the organ. He averred that general depletion could be rarely necessary, and that those who used the greatest depletion were the least successful practitioners.

"I have not seen purulent ophthalmia, whether ordinary or gonorrhœal, treated on this plan;* nor am I aware that any case of the latter kind is recorded. Destructive or injurious consequences have so frequently resulted under the usual management of this disease, that I should certainly employ the local astringent if I met with a case favourable for the trial; that is, where the affection had not extended beyond the conjunctiva. Blood-letting might be resorted to at the same time. In most cases, however, our aid is not sought until the cornea has become affected, and it is therefore too late for the astringent plan. The numerous unfavourable results must be principally ascribed to this circumstance; for, when we see the disease at an early period, we arrest its progress, in a large proportion of instances, by the ordinary antiphlogistic treatment. Six such cases are related in this volume; namely, CASES V., VIII., IX., XI., XII., XIII.; and loss of the eye occurred only in one, CASE V." 46.

A circular incision through the swollen conjunctiva, and excision of the chemosis to let out the gonorrhœal matter have been recommended. The latter would be impracticable in most cases.

Mercury has been freely given. In a case communicated to our author by Mr. Macilwain a high degree of mercurial influence was produced; the eye first affected was lost, the second was saved. Dr. Hennen treated three cases with large bleedings and mercury, affecting the system in forty-eight hours. The event of all was perfectly successful. Depletion was very freely employed in Mr. Macilwain's case, and hence there is some degree of doubt as to which was effective, the bleeding or the mercury, or both. Mr. L. has seen both the ordinary purulent and gonorrhœal ophthalmia proceeding apparently unchecked, under the full mercurial action. Beer and Delpech's experience go to the same conclusion.

The re-production of the urethral discharge supposed to be suppressed, has been a prominent object with some foreign surgeons. Richter even goes so far as to recommend the inoculation of a fresh gonorrhœa, by introducing into the urethra a bougie smeared with gonorrhœal discharge. These are points of practice which will probably receive more smiles than encou-

* "Since the statement in the text was written, I have employed the caustic solution in two cases of conjunctival inflammation with the best result. One of these (CASE XVI.*) was mild gonorrhœal inflammation. The other was catarrhal inflammation of the membrane affecting both eyes of a gentleman, who had been convalescent from gonorrhœa for a few weeks. As he was a person of robust make and full habit, and the eyes were very red and stiff, I took three pounds of blood from the arm, and purged him freely, with relief of the local symptoms, which were completely removed by a subsequent application of the caustic solution. A slight return of redness, without increased secretion took place three days after, in consequence of imprudent exertion of the organs, and a visit to the theatre; it went off without any further treatment."

ragement in this country, and we need not stop to consider them more fully. So much for the chapter on acute gonorrhœal ophthalmia, and its treatment. There is no remarkable novelty in the former, nor any particular precision in the latter. Mr. Lawrence has furnished a catalogue of the modes of practice of others, but with such an extensive experience as he must have had, we looked for more important precepts of his own. His chief reliance appears to be in depletion; he seems to have tried little else. Now if one thing is rendered more plain than another by modern experience, it is this, that general depletion for local affections may be carried too far. This is especially the case with regard to the eye. It is surprising what stimulation it will bear, and that with decided advantage. The employment of applications of astringent or stimulating properties constitutes one of the most important improvements in the treatment of ophthalmia; it has saved many an eye, and will save many a life. If many surgeons have looked upon this modern practice with doubt, their caution is not only excusable but commendable. But when its benefits are pointed out again and again by practical men, it becomes their duty and interest to put it to the test of wide experience. We think then that Mr. Lawrence has hardly experimented so much as he might, with the local application of powerful astringents in gonorrhœal ophthalmia. His remarks, however, are characterised by judgment and discretion.

Twenty four cases of gonorrhœal ophthalmia are related by Mr. Lawrence. We cannot of course give the whole, for although each is separately a valuable fact or series of facts, yet, when another and another still succeeds, like the shades of Scotland's kings, the monotony becomes somewhat tiresome. We do not quarrel with our able author for detailing these cases so fully; on the contrary, we are convinced that if the plan were generally adopted by good and practical writers, the results would be beneficial to the progress of our science. We regret that it does not fall within the scope of a periodical publication to notice such cases as fully as might be wished. We shall select some of those which appear to be most apposite and instructive. And first, of acute gonorrhœal ophthalmia.

Case I. *Gonorrhœal Ophthalmia—Sloughing of the Cornea—Staphyloma Racemosum.*

Mr. M. æt. 24, a stout young man, of full habit, and florid complexion, contracted gonorrhœa in July, 1827. He was not aware of having applied any gonorrhœal matter to the eye. On the 11th of August, he began to feel the right eye uncomfortable; it was more so on the 12th; and his medical attendant recommended the application of brandy and water. The discharge from the urethra had diminished. He went to another practitioner, who bled him on the 12th, 13th, and 14th; applied 60 leeches to the eye; and purged him, &c. He appeared to go on well till the 15th, when he had burning heat and violent pain in the eye and over the brow. On the 16th Mr. Lawrence saw him.

"The eye-lids, particularly the upper, are swollen and red, but they can be opened sufficiently to give a view of the eye. The conjunctiva oculi forms a tumid ring, covering the circumference of the cornea: the latter, though free from ulceration, sloughing, or suppuration, as far as it can be seen, does not possess its natural brilliancy. There is copious yellow discharge from the eye, which is in great pain, as is the head also. He keeps his room totally darkened, finding a great aggravation of the local suffering from even the smallest admission of light. The urethra still produces a whitish discharge in small quantity. I gave a very unfavourable prognosis founded on the constitution and previous state of the patient, as well as on the violence and duration of the present attack, the severe pain in the eye and head, and the condition of the cornea; the latter circumstances particularly made me anticipate the occurrence of serious mischief. I ordered that 20oz. of blood should be taken from the temple by cupping; that active purgatives should be given, and poppy fomentations used to the eye. In the evening, I found that the pain had been relieved by the cupping; the purgatives had not operated; the tongue was foul; there was head-ache and sickness. (Liq. antimon. tart. ℥ss. to be taken immediately, and repeated every hour, so as to produce full vomiting. Twenty leeches afterwards. Cold saturnine lotion to the eye.)" 62.

Next morning twenty-five leeches were applied. The lids and conjunctiva were less swelled—the cornea clear, but not so brilliant as natural—the pain in the head and eye less. The purgatives, not having acted, were repeated freely. On the 18th, the eye was better, the conjunctiva of pale red, the cornea clear, as far as it could be seen. *Twenty leeches—lotion—aperients.* On the 19th the pain was still less—the chemosis had partially subsided, shewing deep ulceration of uncertain extent at the margin of the cornea. On the 20th there was some excitement, with a foul tongue. The ulceration had extended round the entire circumference of the cornea, and through its substance forming a deep trench, on the sides of which, at some points, the corneal laminæ were visible. The part insulated by the circular ulceration was becoming opaque and apparently loosened in texture; it looked like a vesicle or mass of jelly in the front of the eye. *Twelve leeches—saturnine lotion—aperients.* On the 21st there was increased opacity of the insulated cornea, with no pain in the eye. *Alum lotion,* gr. ij. *ad* ℥j.—*soft rag dipped in it applied externally. Infus. ros. c. acid. sulph. dil,* 6*tis hor. Aperient pill—light nutritious diet.* On the 23d, the cornea had become dull, muddy, and opaque throughout, apparently dead—the iris protruded at one part of the ulcer. Vision was irrecoverably gone, and the gentleman was sent into the country for his health.

When this gentleman returned to town, after an absence of several weeks, a protrusion of the iris en masse formed a considerable irregular dark tumour on the front of the globe *(staphyloma racemosum)*, the basis of which corresponded to the circumference of the cornea, while its surface, though divided into smaller prominences, was smooth from being covered by the membrane of the aqueous humour. The friction of this unnatural protrusion irritated the lids and kept up increased redness of the conjunctiva, with slight discharge. Although the greatest attention had been paid to diet, air, exercise, and the regulation of the bowels, they remained costive; the tongue was foul, and the slightest causes would bring on heat, flushing, and pain in the head. The left eye was weak and irritable, and would not bear much employment. In July Mr. Lawrence removed the staphylomatous tumour by passing a cataract knife through its basis. The wound healed and the globe shrunk in the orbit. The left eye was cured by this operation on the right. An artificial eye caused great irritation, and the patient abandoned its use.

Case II. (IV.) *Acute Gonorrhœal Ophthalmia of both Eyes—Disorganization, (general suppuration?) of the Corneæ, and total Blindness.*

This case is short and cannot well be abridged; it illustrates the dreadful effects of neglected gonorrhœal ophthalmia.

"James Ottway, æt. 27, a stout young man, of full habit, and very florid colour, whose occupation had been that of an agricultural labourer, was admitted into St. Bartholomew's Hospital on the 16th of March, 1826, exhibiting, in both eyes, the most destructive effects of acute gonorrhœal inflammation. Five weeks ago, his eyes became sore, and felt as if dust had got into them; the pain increased during the night, which was passed without rest, and the next morning he washed the eyes with his own urine, having been informed by a neighbour that it was a very efficacious remedy. The inflammation grew more and more violent, vision was soon destroyed, and he was obliged to keep his bed.

I found the conjunctiva both of the lids and globe greatly swollen, bright red, raised into irregular prominent masses, and generally granulated. The front of each eye-ball presented a mass of granulations without any appearance of cornea. The tumid conjunctiva had everted both lids of the right eye, and the left superior one, so that the front of the orbits seemed occupied by fleshy masses. There was a copious thin yellow discharge. I could not doubt, on the first view, that it was a case of gonorrhœal ophthalmia, and proceeded to put some questions on the point, when he pretended not to know that there was such a disease as clap. However, on examining the penis, we found discharge from the urethra, warts, and incomplete phymosis; and he acknowledged that he had contracted gonorrhœa five months ago.

As he was of full habit, and had frequent head-ache, he was cupped three times on the back of the neck, had leeches applied to the temples, and was freely and regularly purged, being confined at the same time to milk diet. A solution of alum, gr. iv. to ℥i., was frequently applied to the diseased conjunctiva; and afterwards the undiluted liquor plumbi subacetatis was dropped into the eyes night and morning. He was discharged on the 24th of April, much improved in health, and completely relieved from the ectropium, the swelling of the lids, and the puriform discharge, but in a state of hopeless blindness." 71.

Case III. (V.) *Acute Gonorrhœal Ophthalmia—Opacity of the entire Cornea, and adhesion between it and the Upper Eyelid.*

Mr. D. W. æt. 24, a medical student, had laboured under a slight gonorrhœa for about a week, when in syringing the eyes of an infant labouring under purulent ophthalmia the fluid rebounded into his own right eye. On the third or fourth evening his eye would not bear the bright light of a lecture-room. He had severe pain in the eye during the night, and next morning Mr. Lawrence saw him. The eye-lids were a little red and slightly swollen with serous effusion, while the conjunctiva scleroticæ was raised apparently by the same cause into a pallid red and semi-transparent chemotic ring round the cornea. The blood-vessels of the conjunctiva were little distended. The patient said nothing of the gonorrhœa, and Mr. Lawrence considered it purulent ophthalmia. *Active purge—V.S. to fainting—tartar emetic to keep up vomiting.* In the evening the pain in the eye was worse, and a thin purulent discharge had commenced. On the next morning the palpebral swelling was increased, the eye could not be seen, and there was copious yellow discharge, with severe pain. *V. S. repeated twice—cupping on the nucha and temple—leeches in large number—antiphlogistic regimen.* Each time the blood flowed Mr. W. felt free from pain, but in spite of all the inflammation proceeded. The upper lid was enormously swollen, and a profuse discharge of yellow matter ran in streams over the cheek and temple. After a time astringent lotions were used, and the discharge and swelling slowly abated; but when the palpebræ could be opened the cornea was found to be rather prominent, opaque throughout, and presenting somewhat opaque projections as if new matter had been deposited on it. The conjunctiva of the upper lid adhered to this prominent and irregularly-shaped cornea by a thick broad triangular fold, the apex of which was fixed to the cornea. The eye remained exceedingly irritable, and ultimately Mr. L. was obliged to divide the band in question, and remove the protuberant cornea and iris, as in the operation for staphyloma. Conjunctival inflammation immediately followed, with intolerable pain—opium was required—the tunics collapsed—and an artificial eye could be worn.

CASE IV. (IX.) *Acute Gonorrhœal Ophthalmia—Partial Slough of the Cornea and Prolapsus Iridis—Complete Recovery of Sight.*

R. C. æt. 21, an athletic pugilist, accustomed to eat much animal food, and to drink fermented liquors, contracted a gonorrhœa in the early part of Sept. 1827. In rather more than a fortnight his left eye became inflamed and painful, and probably the discharge was applied to the eyes. On the 20th he was admitted into St. Bartholomew's with acute gonorrhœal inflammation of the eye, the chemosis nearly covering the cornea. *Twelve leeches to the eye—aperients—milk diet.* 21st. Bowels not opened—pulse 96, full and strong—ophthalmia not checked. *Arteriot. temporal. ad* ℥xvj.—*purges.* On the 22d no relief. *V. S. ad syncopen,* (44 *oz.*)—*hirud.* xij. *oculo, calomel and colocynth.* On the 23d and 24th, twenty leeches—clyster on the 25th—bowels opened on 26th by mag. sulph. The pain was severe, the local inflammation continued, and cupping to 20 oz. was practised on the left temple. On the 27th the chemosis and pain were rather less—pulse still powerful. *V. S. to fainting,* (36 *oz.*)—*blister behind the left ear, dressed with savine—aperient mixture.* On the 28th he felt better; the eye was always relieved by depletion. *V. S. to fainting,* (36 *oz.*) On the 29th the cornea could be seen; it was rather hazy; on its lower margin a small vesicle, a protrusion of the membrane of the aqueous humour at an ulcerated aperture, probably produced by the separation of a slough. *Blister to the temple.* Oct. 1st, still profuse discharge. *Solut. zinc. sulph.* (gr.j. ad ℥j.) *sæpius injiciend.* On the 2d, the cornea was clear—prolapsus iridis had taken place at the ulcer, which involved only a small portion of the pupillary margin. On the 6th, this, covered by the membrane of the aqueous humour, formed a smooth oblong tumour on the margin of the cornea, one-third of an inch in length, and half as broad. The patient got perfectly well, and vision was unimpaired. In 1829, there was a nearly circular opacity of the cornea, one-eighth of an inch in diameter, in the site of the protrusion; the iris adhered to it.

CASE V. (XI.) *Acute Gonorrhœal Ophthalmia—Deep Ulcer at the Margin of the Cornea—Complete Recovery.*

John Biggs, æt. 21, had been subject to sore eyes, when he contracted a gonorrhœa rather severe. In 11 weeks the right eye began to inflame. In another week he was admitted into the Ophthalmic Infirmary. The lids were tumid; the chemosis concealed the circumference of the cornea, the centre of which was nearly clear; there was severe pain and profuse discharge; the gonorrhœa continued, with some ardor urinæ. *Arteriot. temp. ad* ℥xvj.—*hirud.* xij.—*calom. et jalap.—mag. sulph.* 6*tis hor.*—*Vesicat inter scapulus.* The leeches and blisters were repeated, and soon an alum lotion was substituted for a saturnine. As the chemosis subsided, a deep ulcerated trench was seen at the margin of the cornea, extending for about five-sixths of its entire circumference; within this trench the cornea was slightly nebulous. The ulcer proceeded favourably, and in a month after his admission the patient was dismissed with perfect vision. The ulcer healed, and left merely a slight white line near the edge of the cornea.

We have only room for one other case of acute gonorrhœal ophthalmia, in which mercury was employed to its full extent by Mr. Macilwain. It is not susceptible of abbreviation. The patient was a female.

CASE VI. (XIV.) *Acute Gonorrhœal Ophthalmia—Recovery of one Eye—Opacity of the Cornea, Adhesion of the Iris, and Loss of Sight in the other.*

" 'The patient, a young woman, applied to me (says Mr. Macilwain) for what she called weak eyes, which I found, on examination, to be a slight degree of catarrhal ophthalmia. I merely ordered her some aperient medicine and poppy fomentation, and, in a few days, the disease had nearly disappeared. After an interval, however, of about three days, she again applied, when I was surprised to find both eyes affected by violent gonorrhœal inflammation. The palpebræ were much swollen; the conjunctiva lining them, as well as that covering the globe, in a state of acute inflammation, the latter portion of the membrane presenting a much raised chemosis; the cornea dusky; great pain in the eyes and head, with a profuse purulent discharge. I now learned, for the first time, that she had gonorrhœa; but I was, for some minutes, foiled in my endeavours to elicit from her any circumstances to justify the opinion that the vaginal discharge had been applied to the eyes; and, when interrogated on that point, she said that she had been particularly cautious in avoiding the use of any linen for other purposes, which had been employed in cleansing the parts affected with gonorrhœa. As she was about to leave the room, her mother reminded her that since I last saw her she had washed her eyes with her own urine, and that they had become much worse immediately afterwards, the pain increasing with the addition of discharge and the other symptoms, of which she now complained. The treatment consisted in the free abstraction of blood, and the administration of brisk saline purgatives every three hours till the bowels had been largely evacuated. She was bled from the arm ad deliquium three times, and blood was afterwards taken locally by cupping and leeches. One grain of calomel with a quarter of a grain of opium was then prescribed thrice a day. The effect of this was that one night, without previous warning, she was seized with the most violent degree of ptyalism I ever witnessed. The tongue became prodigiously swollen, hanging out of the mouth, and presenting an appearance which induced apprehensions of its sloughing. The mercury was discontinued, the only other measures employed being the application of belladonna round the palpebræ, and frequent ablution, with poppy fomentation. The result of the case was complete restoration of one eye, partial opacity of the cornea in the other, with adhesion of the closed pupil, and loss of sight.'

Mr. MACILWAIN adds, 'I always employ mercury in the treatment of this disease; not from any opinion of the identity of the gonorrhœal with the syphilitic virus, but from it having appeared to me that the degree of recovery in such cases had some relation to the severity of the new action produced by this mineral. In following this plan, I have been further influenced by the too frequent inefficiency of the most active anti-inflammatory treatment, when employed alone.' " 104.

We shall now relate two cases of the mild gonorrhœal inflammation of the conjunctiva; in the first there is no arthritic affection, in the second there is.

CASE VII. (XVI.) *Gonorrhœa, with mild Inflammation of the Conjunctiva.*

Mr. E. L., æt. 26, having never suffered from rheumatic symptoms, had simple gonorrhœa. Two years after this he contracted another, and in five or six days his eyes became inflamed. On the 8th the palpebral conjunctiva was bright scarlet; on the circumference of the globe it was of the same colour, but on the anterior part of the sclerotica it was not much affected. The sclerotica did not participate in the inflammation. A thick portion of thick white mucus lay between the lower lid and the globe on each side, and a little yellow discharge appeared at the inner angle of each eye. There was slight general swelling of the lids, and the eyes were watery, without intolerantia lucis. *Saturnine lotion—aperient—salines with antimony.* On the 10th day the eyes were nearly in the same state. *Solut. argent. nit.* (gr. iv. ad ℥j.) *dropped into each. Collyrium of hyd. oxymur.* gr. j. *in aq. ros.* ℥viij. *Ointment of hyd. nit. oxyd.* gr. ij. *fresh butter* ʒi. *applied to edges of lids at night.* The nitrate of silver wash caused some smarting at first, but by a repetition of this treatment the eyes became perfectly well, and so continued. A slight gleet remained.

CASE VIII. (XVIII.) *Gonorrhœa—Affection of the Joints—mild Inflammation of the Conjunctiva.*

Mr. C., æt. 38, a corpulent free eater, subject to rheumatism, contracted gonorrhœa. Subsequently the synovial membrane of the right knee became inflamed, and there was general and painful swelling of the hands. When Mr. Lawrence saw him the knee-joint was diminishing. *Plummer's pill—colchicum—evaporating spt. lotion.* The gonorrhœal discharge ceased, and the joints became better. At this time, mild inflammation of the conjunctiva came on in both eyes, and went off in three or four days, with tepid lotions and a continuance of the former medicines. After the application of a blister to the knee the effused fluid was completely absorbed: the hands remained slightly puffed and tender, but these symptoms soon disappeared, under the use of warm sea bathing and Plummer's pill.

The last few cases in Mr. Lawrence's ample porte-feuille, are examples of the gonorrhœal inflammation of the external tunics of the eye.

CASE IX. (XXI.) *Acute Inflammation of the External Tunics, with Gonorrhœa —Rheumatism.*

A medical gentleman, æt. 24, of rather delicate conformation, contracted a severe gonorrhœa. In three weeks the eyes became inflamed without obvious cause; they were red, very painful, acutely sensible to light, and watered copiously; there was some mucous discharge. He lost 150 ozs. of blood from the arm and had 230 leeches applied to the eyes which recovered completely. The urethral discharge had previously diminished; it ceased altogether during this active treatment. In twelve or eighteen months another attack occurred after gonorrhœa of a fortnight's growth. The conjunctiva suddenly became inflamed without obvious cause, he lost 18 ozs. of blood, and the membrane became clear. Next morning there was a relapse, arrested by 12 leeches. The gonorrhœa now got nearly well, the patient went into the country, and took cold from wet feet. The eye became again affected, but in a different way; there was great pain, excessive intolerance of light, profuse watering; the sclerotica and iris were affected. *V.S. brach. ad* ℔. iv. *in three days*—100 *leeches—colchicum and counter-irritation.* There was little or no benefit, when a metastasis took place from the eyes to the limbs every joint becoming more or less affected, and the right knee greatly distended. The strength was, naturally enough, much reduced. The affection of the eyes and limbs alternated for a fortnight, when the former became quite well, but the patient remained crippled. He was now put on tonics, with sea air, warm salt bathing, and friction. Under this plan he was cured, and remained well till the Winter of 1828, when he had severe affection of the joints from cold. The eyes did not suffer. He recovered completely.

CASE X. (XXIII.) *Gonorrhœa—Rheumatism—Gonorrhœal Inflammation of the External Tunics.*

Mr. F., æt. 29, had not suffered from rheumatism, with the exception of some slight occasional pains, and had once had a mild gonorrhœa. On the 14th of December, 1829, Mr. Lawrence was called to see him. He had laboured under gonorrhœa for seven weeks, had felt severe pains in the back and lower limbs for a fortnight. The eyes had become sore on the 13th, and on the morning of the 14th he had been bled from the arm to forty ounces with relief. There was still considerable distention of the sclerotic of both eyes, pain, particularly in the right, intolerance of light, and profuse lachrymation. *Poppy fomentations—aperients—salines with antimonials.* On the 15th the patient complained of considerable pain in the right eye, which was most inflamed. *Twelve leeches to the right eye—eight to the left.* After this the eyes were nearly well, and the urethral discharge increased. The patient took great care of himself, yet on the 27th of January the eyes grew worse. On the 28th he was seen by Mr. Lawrence. The inflammation had returned—there was a red zone round each cornea—a small white speck near the centre of the left—considerable pain—profuse lachrymation and dimness of vision, especially in the left eye. *Twenty leeches—blister to the nucha—calomel and jalap followed by salts—poppy fomentation.* The eyes were much better the next day. *Plummer's pill.* On the 6th Feb. the eyes appeared quite well, and the vision was clear. On the 20th he had troublesome rheumatic pains, confined to the lower extremities, and chiefly affecting the left hip and heel, and the right instep. *Plummer's pill continued—colchicum.* The gentleman soon removed to the sea side, and took warm sea bathing, and at the end of a month he returned to London and resumed his usual avocations.

We have not space for the insertion of other cases. In the next article we shall pass to the second portion of the volume, which treats of the syphilitic diseases of the eye. We cannot part from Mr. Lawrence without warmly thanking him for the pleasure we have experienced in perusing the present work. The subjects are treated with clearness and talent, and we recommend the book to the attentive consideration of every practical surgeon.

